# Citrate anticoagulation for continuous renal replacement therapy in small children

**DOI:** 10.1007/s00467-013-2690-6

**Published:** 2013-12-13

**Authors:** Jolanta Soltysiak, Alfred Warzywoda, Bartłomiej Kociński, Danuta Ostalska-Nowicka, Anna Benedyk, Magdalena Silska-Dittmar, Jacek Zachwieja

**Affiliations:** 1 Department of Pediatric Cardiology and Nephrology, Poznan University of Medical Sciences, Poznan, Poland; 2 Department of Pediatric Cardiac Surgery, Chair of Cardio-Thoracic Surgery, Poznan University of Medical Sciences, Poznan, Poland; 3 Department of Pediatric Cardiology and Nephrology, Cardio-Thoracic Surgery, Poznan University of Medical Sciences, 27/33 Szpitalna St., 60-572 Poznan, Poland

**Keywords:** Anticoagulation, Citrate, Children, Renal replacement therapy

## Abstract

**Background:**

Regional citrate anticoagulation (RCA) is one of the methods used to prevent clotting in continuous renal replacement therapy (CRRT). The aim of this study was to describe the outcomes and complications of RCA-CRRT in comparison to heparin anticoagulation (HA)-CRRT in critically ill children.

**Methods:**

This study was a retrospective review of 30 critically ill children (16 on RCA- and 14 on HA-CRRT) who underwent at least 24 h of CRRT. The mean body weight of the children was 8.69 ± 5.63 kg. RCA-CRRT was performed with a commercially available pre-dilution citrate solution (Prismocitrate 18/0).

**Results:**

The mean time on RCA-CRRT and HA-CRRT was 148.73 ± 131.58 and 110.24 ± 105.38 h, respectively. Circuit lifetime was significantly higher in RCA-CRRT than in HA-CRRT (58.04 ± 51.18 h vs. 37.64 ± 32.51 h, respectively; *p* = 0.030). Circuit clotting was observed in 11.63 % of children receiving RCA-CRRT and 34.15 % of those receiving HA-CRRT. Episodic electrolyte and metabolic disturbances were more common in children receiving RCA-CRRT. The survival at discharge from the hospital was 37.5 and 14.3 % among children receiving RCA-CRRT and HA-CRRT, respectively.

**Conclusions:**

In critically ill children with a low body weight, RCA appeared to be safe and easy to used. Among our patient cohort, RCA was more effective in preventing circuit clotting and provided a better circuit lifetime than HA.

## Introduction

Continuous forms of renal replacement therapy (CRRT) have become established as the treatment of choice for supporting critically ill patients with acute kidney injury [1, 2]. These patients present with activation of the coagulation cascade, peripheral mononuclear cells and platelets, as well as a reduction in natural anticoagulants; consequently, they are prothrombotic [3]. Regional anticoagulation with citrate (RCA) is one method used for preventing clotting in the CRRT circuit. The advantages of RCA include a better circuit lifetime as compared to unfractionated heparin anticoagulation (HA) and reduction of heparin-induced thrombocytopenia. The latter is of particular relevance in patients with a high risk of bleeding. A number of studies have reported that HA-CRRT is associated with a reduction of bleeding due to regional anticoagulation, which applies only to the circuit, and a lack of systemic anticoagulation [3–6]. In contrast, a recent meta-analysis of 488 patients did not show any difference between heparin and citrate in terms of circuit life with the main difference between the two modalities being a decrease in bleeding complications with citrate [7]. However, there was a significant heterogeneity in the primary outcome of the studied group. Citrate can provide anticoagulation of blood since it binds with calcium and renders calcium unavailable to the clotting cascade. The absence of calcium prevents clotting. Nevertheless, excess citrate may lead to metabolic complications (acidosis or alkalosis) and electrolyte disturbances, especially hyper- and hyponatremia, and hyper- and hypocalcemia [3–5].

 Only a few studies have reported RCA-CRRT use and outcomes in children. The aim of this study was, therefore, to describe the clinical application, outcome and complications of RCA-CRRT in comparison to HA-CRRT in critically ill children.

## Methods

A retrospective review was conducted in 30 children (mean body weight 8.69 ± 5.63 kg) who had received at least 24 h of continuous arterio–venous or venous–venous hemodiafiltration in the University Hospital, Poznan, Poland, between January and December 2012. HA was used as the standard anticoagulation method (*n* = 14) at the University Hospital until the middle of 2012 when it was replaced by RCA which was used thereafter in the treatment of new patients (*n* = 16). No special selection or randomization was used. Disease categories in these children included nephrotic syndrome (2 patients), multiorgan dysfunction due to cardiac surgery (18 children), sepsis (3 children), leukemia (3 children) and metabolic diseases (2 children). CRRT was started due to clinical features and the presence of life-threatening edemas despite enhanced diuresis. Therefore, in some cases, CRRF was started before the failure stage according to pediatric RIFLE (Risk, Injury, Failure, Loss, End-stage kidney disease) criteria [8]. Children on RCA-CRRT did not differ from those on HA-CRRT in terms of age, body weight and creatinine level. Initial clinical and laboratory data are given in Table 1.

**Table 1:** Clinical characteristics of patients at the time of initiating regional citrate anticoagulation- and heparin-anticoagulation renal replacement therapy

Clinical characteristics	RCA-CRRT	HA-CRRT	*p*
No. of patients	16	14	na
Gender (*n*)	7 females/9 males	8f/6m	na
Disease categories (*n*)	CS (11)/S (3)/NS (2)	CS (7)/LEU (3)/MODS (2)	na
Age (months)	15.25 ± 24.10	24.15 ± 27.96	0.523
Weight (kg)	7.76 ± 4.67	9.77 ± 6.21	0.355
Mean arterial pressure (mmHg)	72 ± 27.59	66 ± 12.86	0.938
Edema (%)	100	85	na
Mechanical ventilation (%)	75	86	na
Total parenteral nutrition (%)	75	93	na
Multiorgan dysfunction syndrome (MODS) (%)	75	93	na
Creatinine (mg/dl)	1.04 ± 0.66	0.93 ± 0.79	0.294
Blood urea nitrogen (mg/dl)	103.56 ± 70.61	96.64 ± 62.72	0.822
Aspartate aminotransferase (IU/l)	1828.67 ± 3230.12	592.64 ± 1096.29	0.354
Acute liver failure (*n*)	8 (50 %)	6 (43 %)	na
Systemic sodium (mmol/l)	144.82 ± 8.67	142.06 ± 8.89	0.377
Systemic ionized Ca (mmol/l)	1.11 ± 0.18	1.10 ± 0.17	0.892
Systemic lactate (mmol/l)	6.44 ± 4.82	5.15 ± 5.35	0.492
pH (units)	7.39 ± 0.13	7.41 ± 0.08	0.983
HCO_3_ (mmol/l)	21.46 ± 5.54	25.02 ± 6.14	0.387
Base excess (units)	−3.68 ± 6.85	0.28 ± 6.75	0.467
Activated partial thromboplastin time (s)	44.47 ± 16.69	55.09 ± 68.36	0.224

RCA, Regional citrate anticoagulation; HA, heparin anticoagulation; CRRT, continuous renal replacement therapy; CS, after cardiac surgery; S, sepsis; NS, nephrotic syndrome; LEU, leukemia; na, non-applicable

Unless indicated otherwise, data are presented as the mean ± standard deviation (SD)

Ethics Committee approval was not required for this observational study because all data reported, as well as anticoagulation method assignment, are part of our routine medical procedures and guidelines.

CRRT was performed using the Prismaflex system (Gambro, Lundia AB, Lund, Sweden) including the Prismaflex HF-20 or —60 hemofiltration system (Gambro). Vascular access varied based on the size of the child. In neonates weighing <5 kg, two central catheters were used with minimum size of 20G for arterial access and 18G for venous access. In older children double-lumen accesses were used.

RCA-CRRT was performed using a commercially available pre-dilution citrate solution (Prismocitrate 18/0; Gambro) and a commercially available dialysate normocarbonate solution (Prism0cal B22; Gambro), according to the Intensive Care Units Therapeutic Recommendation for CRRT by Gambro, which is based on the recommendations of Tolwani et al. [9]. Prismocitrate 18/0 consists of 0.5 % trisodium citrate solution, which contains 18 mmol/l citrate without citric acid and 140 mmol/l sodium. In relation to blood flow rate, the citrate solution rate was set to achieve a circuit citrate concentration of 3 mmol/l blood flow. The dosage of citrate in millimoles per liter blood flow was defined as the pre-dilution citrate load. The pre-dilution citrate load was modified, if needed, to obtain circuit ionized calcium in the range 0.25–0.50 mmol/l (post-filter sample). To assess the dosage of citrate in millimoles per hour delivered to the patient, we calculated the estimated citrate load. The post-dilution bicarbonate solution rate was adjusted to obtain a total dialysis dose of >35 ml/kg/h. Calcium glubionate and calcium gluconate (10 and 5 % solution, respectively, in children weighing <10 kg) were infused in the separate central catheter if a double-lumen catheter was used or in a venous single catheter. Calcium compensation was defined as a percentage of supplemented calcium solution when compared to calcium loss with dialysate, and it was set to the machine according to systemic calcium. Calcium solution flow was calculated automatically based on pre-dilution citrate load, dialysate flow and type of calcium solution used. Systemic ionized calcium was maintained in the normal range from 0.9 to 1.2 mmol/l.

In patients with HA-CRRT the conventional heparin protocol was applied as follows: mean initial bolus of unfractionated heparin was in the range 20–30 IU/kg and subsequent continuous infusion of heparin ranged from 10 to 20 IU/kg/h, adjusted to achieve the target activated partial thromboplastin time (APTT) ratio of approximately 1.5. PrismaSol 2 (Gambro) was used as a dialysate and replacement fluid. Blood flow rate (BFR) was determined as 2–3.6 ml/kg/min and ranged from 16 to 24 ml/min. The recommended minimum BFR for the smallest HF20 filter is 20 ml/min. In the children included in our study, it was very difficult due to small-sized catheters to obtain a higher BFR. Hypotension, which is one of the complications that can arise at the time the patient is being connected to the hemodiafiltration system, especially cardiac patients, was defined as a fall in the mean blood pressure of >20 mmHg relative to baseline during the first 60 min after connection to the CRRT system. To avoid hypotension, the circuits were primed with 5 % albumin solution or with blood. Multiorgan dysfunction syndrome (MODS) was defined as the presence of at least three failed organs. Organ system failure was defined using the international pediatric sepsis consensus conference definitions [10]. The presence of acute liver failure was defined as the abrupt loss of liver function without pre-existing liver disease and by determining laboratory liver function parameters [elevated aspartate aminotransferase and coagulopathy with a prothrombin time of ≥15 s or an international normalized ratio of ≥1.5]. Due to general anesthesia, the encephalopathy criterion was not considered [11]. Electrolyte disturbance during CRRT was as categorized as follows: hypocalcemia (ionized systemic calcium <0.9 mmol/l), hypercalcemia (ionized systemic calcium >1.2 mmol/l), hyponatremia (sodium <129 mmol/l), hypernatremia (sodium >143 mmol/l), metabolic acidosis (pH <7.35, HCO_3_ <22 mmol/l) and metabolic alkalosis (pH >7.45 and HCO_3_ >26 mmol/l). The risk of bleeding in patients who had undergone cardiac surgery was assessed based on blood transfusion rate (ml/kg/day).

Statistical analysis was performed using Statistica ver. 8 (StatSoft, Tulsa, OK). The data are reported as the mean ± standard deviation. Normally distributed continuous variables were tested using the analysis of variance with Tukey post-hoc test. In all other cases, non-parametric tests were applied using the Mann–Whitney and Kruskal–Wallis tests. Circuit lifetime was assessed using Kaplan–Meier survival analysis, and survival curve distribution was compared with the log rank (Mantel–Cox) test. The level of significance was set at *p* < 0.05.

## Results

The mean time on RCA-CRRT and HA-CRRT was 148.73 ± 131.58 and 110.24 ± 105.38 h, respectively. In total, 43 circuits were used in RCA-CRRT, with a filter life of 58.04 ± 51.18 h (median 39.75 h, range 1–171.8 h, total 2,379.65 h); when scheduled CRRT shutdowns were excluded, the mean filter life was 65.31 ± 53.90 (median 72.25) h. Only five circuits (11.63 %) had to be shut down due to filter clotting. The most common cause of circuit shutdown was associated with the handling of system alarms (Table 2). At the time of initiating RCA-CRRT the pre-dilution citrate load was always 3 mmol per liter of blood flow using the Prismocitrate 18/0 solution. In 15 children initial calcium compensation was 100 %; only in 1 case, due to elevated systemic calcium, was calcium concentration reduced to 90 %. The initial parameters of both RCA-CRRT and HA-CRRT are given in Table 3. Total dialysis dose in all children achieved the recommended level, and the mean value was 52.32 ± 35.63 ml/kg/h. Target circuit and systemic Ca^++^ were easily maintained and were 0.38 ± 0.05 and 1.17 ± 0.17 mmol/l, respectively.

**Table 2:** Causes of stops in CRRT

Cause of CRRT circuit shutdown	Number of circuits (%)	
	RCA-CRRT	HA-CRRT
Diuresis	8 (18.60)	5 (12.19)
Alarm handling/technical issues	13 (30.23)	8 (19.51)
Medical procedures	6 (13.95)	5 (12.19)
Death	7 (16.28)	7 (17.07)
CC malfunction	4 (9.30)	2 (4.88)
Clotting	5 (11.63)	14 (34.15)
Total number of circuits	43	41

CC, Central catheter; CRRT, continuous renal replacement therapy; RCA, regional citrate anticoagulation; HA, heparin anticoagulation

**Table 3:** Initial parameters of CRRT

Parameters	RCA-CRRT	HA-CRRT
Blood flow rate (ml/kg/min)	3.49 ± 1.56	2.88 ± 0.80
Pre-dilution citrate load (mmol/l blood flow)	3 ± 0.00	–
Estimated citrate load (mmol/h)	4.05 ± 2.30	–
Dialysis dose (ml/kg/h)	52.32 ± 35.63	71.71 ± 39.39
Calcium compensation (%)	99.38 ± 2.42	–
Heparin dose (IU/kg/h)	–	17 ± 10

–, Data not available; CRRT, continuous renal replacement therapy; RCA, regional citrate anticoagulation; HA, heparin anticoagulation

Data are presented as the mean ± SD

**Fig. 1:**
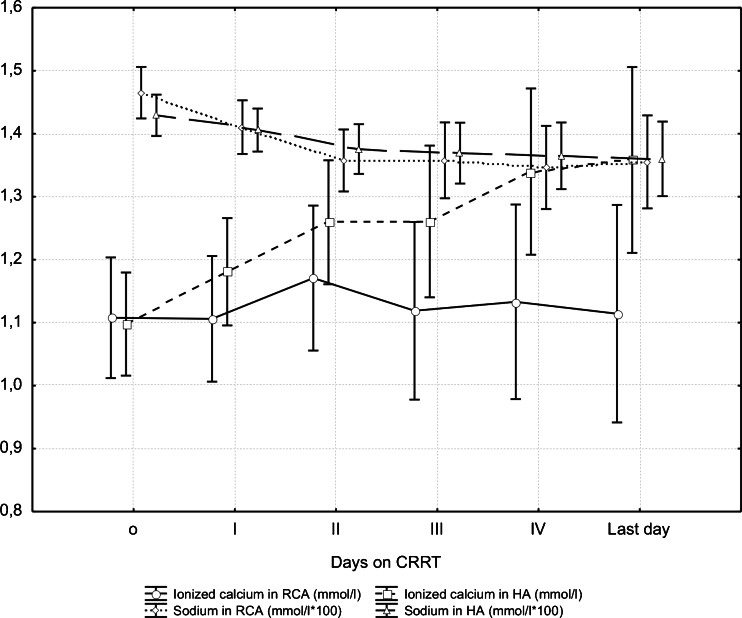
Systemic sodium and ionized calcium loads during consecutive days of regional citrate anticoagulation (*RCA*)- and heparin anticoagulation (*HA*)-continuous renal replacement therapy (*CRRT*). Significant reduction of systemic sodium load on days 2, 3 and 4 versus the initial (pre-CRRT) value (day 0). *p* values were as follows: *p*_2_ = 0.023; *p*_3_ = 0.015; *p*_4_ = 0.005

In HA-CRRT the mean circuit functional survival was 37.64 ± 32.51 h (median 25 h, range 2.25–121 h, total 1,398.33 h). The main cause of circuit shutdown was clotting (34.15 %; see Table 2 for other causes). Analysis of the main metabolic and electrolyte parameters throughout the first 4 days of RCA-CRRT revealed a significant reduction of systemic sodium and potassium levels on days 2, 3 and 4 versus the initial value (pre- CRRT; day 0) (Fig. 1). A similar trend was observed in HA-CRRT, but the differences were not significant (Fig. 1). Hyponatremia was more common in children receiving RCA-CRRT than in those on HA-CRRT (18.75 vs. 0 %, respectively); the same was true for hypokalemia (62.5 vs. 28.6 %, respectively) (Table 4). Episodic mild hypo- and hypercalcemia was more frequent in children on RCA-CRRT, but it was immediately offset by calcium compensation and citrate load. During consecutive days of RCA-CRRT there were no significant differences between the systemic and circuit calcium loads (Fig. 1). The characteristic trend of a reduction in calcium compensation and an increase in pre-dilution citrate load was observed. Calcium compensation was significantly lower on day 4 and on the last day of the therapy than on day 1. The differences in citrate load at these times were not significant.

**Table 4:** Adverse events among the pediatric patient cohort on RCA-CRRT and HA-CRRT

Adverse events	Number of patients (%)	
	RCA-CRRT	HA-CRRT
Hypernatremia (>143.0 mmol/l)	3 (18.75)	2 (14.3)
Hyponatremia (<129.0 mmol/l)	3 (18.75)	0 (0)
Hyperkalemia (>5,8 mmol/l)	2 (12.5)	3 (21.4)
Hypokalemia (<3,6 mmol/l)	10 (62.5)	4 (28.6)
Hypercalcemia (>1.2 mmol/l)	7 (43.75)	9 (64.3)
Hypocalcemia (<0.9 mmol/l)	7 (43.75)	0 (0)
Metabolic acidosis (pH <7.35 and HCO_3_ <22 mmol/l)	7 (43.75)	6 (42.9)
Metabolic alkalosis (pH >7.45 and HCO_3_ >26 mmol/l)	4 (25)	2 (14.3)

RCA, regional citrate anticoagulation; CRRT, continuous renal replacement therapy; HA, heparin anticoagulation

Mild metabolic acidosis occurred in sevent of 16 patients on RCA-CRRT and was treated by infusing sodium bicarbonate into the dialysate fluid (3.06 ± 4.63 mmol/l dialysate). Metabolic alkalosis was observed only in four cases (Table 4).

The blood transfusion rate in children after cardiac surgery did not differ between the two study groups. However, the activated partial thromboplastin time (APTT) was similar in both groups during consecutive days of CRRT, as was the platelet count, although the mean values were higher in the RCA-CRRT group.

**Fig. 2:**
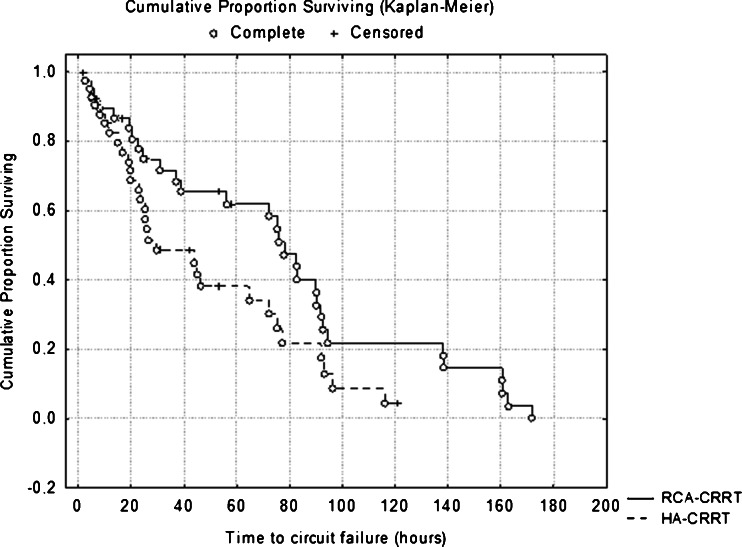
Kaplan–Meier curves of circuit lifetime probability according to RCA- and HA-CRRT. Causes of scheduled CRRT shutdowns have been censored. Survival curve distribution is compared with the log rank (Mantel–Cox) test (*p* = 0.030). RCA, regional citrate anticoagulation; HA, heparin anticoagulation; CRRT, continuous renal replacement therapy

Circuit lifetime was significantly higher for the RCA-CRRT system when compared to the HA-CRRT system (*p* = 0.030). While 41.86 % of the circuits used in RCA-CRRT functioned for longer than 72 h, only 21.95 % of those used in HA-CRRT did so. For each anticoagulation modality, the Kaplan–Meier curves of circuit lifetime probability, derived from our analysis of scheduled and unscheduled CRRT interruptions, are displayed in Fig. 2.

The cessation of RCA-CRRT was due to effective diuresis in nine cases and due to death in seven cases (in HA-CRRT: 5 and 9 children, respectively). The deaths were related to severe disease and MODS. The survival at discharge from the hospital was 37.5 % in the children receiving RCA-CRRT and 14.3 % in those receiving HA-CRRT. At the time of discharge renal function was normal, and no patients required dialysis.

## Discussion

Continuous renal replacement therapy is closely related to the need for efficient anticoagulation. Citrate provides regional anticoagulation that is essentially restricted to the extracorporeal circuit, where it acts by chelating ionized calcium. Our experience and the results of our analysis show that this method is of great value even in small children—but it is not perfect.

First, a certain amount of citrate reaches the systemic circulation where its metabolism predominantly occurs in the hepatic citric acid cycle; consequently, clearance is almost independent of renal function [12, 13]. The metabolism of one citrate molecule releases three sodium bicarbonate molecules and ionized calcium [3, 14]. Therefore, the excess citrate can cause metabolic alkalosis, hypernatremia and hypercalcemia, any of which typically occurs after 36 h of treatment [3, 5, 14].

The most dangerous complication of RCA is systemic hypocalcemia, which may even be life-threatening, especially in cardiac patients [3, 5, 14]. Our analysis of electrolyte disturbances revealed the occurrence of episodic hypocalcemia in seven of our patients (43.75 %) on RCA-CRRT; in comparison, no patient on HA-CRRT developed hypocalcemia. However, in patients on RCA-CRRT, the hypocalcemia was incidental and reached a minimum value of 0.7 mmol/l in one case. The solubilized (s) Ca^++^ was immediately corrected by calcium re-infusion through a separate line, and no side effects in terms of cardiac dysrhythmia related to hypocalcemia were observed. Although 50 % of patients suffered from acute liver failure, no patient developed persistent hypocalcemia, and none required citrate reduction or early cessation of CRRT. The calcium ratio was in all cases below 2.5. Among our patients, the incidental cases of hypocalcemia probably resulted from the dialytic removal of the newly formed calcium citrate and the use of calcium-free dialysate, which is also considered by other authors to be a cause of calcium disturbances [3, 5, 14]. Liver failure does not appear to be a contraindication to RCA. Schultheiß et al. also postulate that RCA would appear to be feasible in patients with severely impaired liver function [15, 16].

Hypercalcemia occurred in 43.75 % of our patients during RCA-CRRT, while in those on HA-CRRT it was more common and persistent (64.3 %). In RCA-CRRT the maximum value of systemic Ca^++^ was 1.4 mmol/l, suggesting that the hypercalcemia was probably due to additional calcium infusion, such as in the total parenteral nutrition (TPN). In our opinion, it is extremely important to control the composition and the rate of infusion of all extra fluids given to the patient. If TPN contains calcium, both the calcium concentration and the rate of infusion should be stable. In our pediatric patients on RCA-CRRT, TPN resulted in a significant reduction of calcium compensation on day 4 and the last day of treatment versus day 1.

An excessive citrate load can cause metabolic alkalosis. To avoid an excessive alkali load, the dialysate fluid contains a normal bicarbonate concentration (22 mmol/l). The hyperchloremic fluids used in this protocol also reduce alkalosis. Therefore, metabolic alkalosis occurred relatively rarely: in only four children on RCA-CRRT and two children on HA-CRRT. A more common adverse event among our patient cohort was metabolic acidosis, which occurred in children on RCA-CRRT and on HA-CRRT. In RCA-CRRT metabolic acidosis can result mainly from the difficulty in citrate metabolism [3, 5]. However, the high dialysate flow and increased bicarbonate loss with effluent can also result in metabolic acidosis. It has been reported that citrate clearance is similar to that of urea and that the metabolic acidosis could result from a too high dialysis dose and loss of bicarbonate with the effluent [17]. The mean value of the dialysis dose in our study was 52.32 ± 35.63 ml/kg/h.

A very important electrolyte disturbance during citrate anticoagulation is hyper- or hyponatremia. The commercially available citrate solutions contain high concentrations of citrate and sodium [3]. The Prismocitrate 18/0 used in this study contained 18 mmol/l trisodium citrate solution, and citrate metabolism resulted in three additional molecules of sodium. However, hypernatremia is uncommon if a hypo- or isonatremic dialysate is used [5]. Our results confirm this: short-lasting hypernatremia occurred only in three patients, usually during the first day of treatment, and was related to elevated initial sodium levels. The characteristic trend towards a reduced sodium concentration during consecutive days of RCA-CRRT was observed, with a significant reduction of the systemic sodium concentration becoming apparent on days 2, 3 and 4 versus the initial value on CRRT initiation (day 0). Although in routine clinical practice, the sodium balance is not only dependent upon potential sodium gains from the citrate solutions but also on the sodium content of other infusions, feeding and sodium losses through urine and drains, our results indicate a citrate anticoagulation-dependent reduction of systemic sodium with a minimum value of 121 mmol/l [3].

The findings of our study show a significantly longer circuit lifespan during RCA-CRRT relative to HA-CRRT that resulted in fewer connection–disconnection procedures. This is the main advantage of using RCA-CRRT as shown in our study. This observation is consistent those of other authors, who reported better filter survival in adults [4, 18–20]. A better circuit lifespan minimizes treatment downtime and reduces the risk of ineffective therapy, blood loss and hemodynamic instability during circuit exchange [21]. This is especially important in very small children who have a low blood volume and who are additionally at a high risk of the bradykinin release phenomenon [22]. The longer circuit lifetime found in this study enhances the known advantages of using RCA-CRRT. We did not compare prescribed versus delivered dose as a measure of efficiency of anticoagulation. The delivered dose of dialysis is a final consequence of different factors, such as actual blood flow, membrane permeability, convection rate, among others, which are sometimes overlying, overlapping and indistinguishable.

The most common cause of circuit shutdown in RCA-CRRT was associated with the handling of alarms, while in HA-CRRT it was associated with clotting. Most of the problems in RCA-CRRT were caused by technical issues, probably due to the small size of the circuits, low blood flow and small catheters. Prolonged circuit lifetime, without filter clotting in any case, has also been reported to be a cause of circuit shutdown in RCA-CRRT [4, 18, 19]. However, these studies were conducted in adults, and the authors targeted a lower post-filter Ca^++^ level (<0.4 mmol/l) than we did in our study. Although we found the mean value of circuit Ca^++^ was 0.38 ± 0.05 mmol/l, the episodic measurements above 0.40 mmol/l might be sufficient to start clotting. Opatrny et al. concluded that the usually recommended circuit Ca^++^ levels do not guarantee complete prevention of hemostasis activation [23]. In another study, the post-filter Ca^++^ level reliably predicted an activated coagulation time of >120 s, which was also affected by citrate when the Ca^++^ targeted level was ≤0.30 mmol/l [24]. Therefore, in our opinion, a target level of <0.40 mmol/l circuit Ca^++^ might better prevent clotting, especially when small circuits with a low blood flow are used.

There were no significant differences in platelet count and blood transfusion rate between patients on RCA- and HA-CRRT. However, the APTT level was similar in both groups during consecutive days of CRRT, and critically ill children with a risk of clotting received systemic heparin. However, in those patients on HA-CRRT, especially in cardiac patients, the dosage of heparin was relatively low to reduce the risk of bleeding, and the mean APTT level was 53 ± 12 s. To assess the effect of citrate on the risk of bleeding, further studies with a larger and homogeneous group of patients are needed.

In summary, the critically ill children with a low body weight in our study, the RCA protocol adopted in our study appears to be safe and easy to used. RCA was more effective in preventing circuit clotting when compared to heparin anticoagulation. The metabolic and electrolyte complications were relatively low and simple to correct even in patients with liver failure. We recommend a target circuit Ca^++^ concentration of between 0.3 and 0.4 mmol/l as sufficient to prevent clotting.
